# Characterizing Public Sentiments and Drug Interactions in the COVID-19 Pandemic Using Social Media: Natural Language Processing and Network Analysis

**DOI:** 10.2196/63755

**Published:** 2025-03-05

**Authors:** Wanxin Li, Yining Hua, Peilin Zhou, Li Zhou, Xin Xu, Jie Yang

**Affiliations:** 1 School of Public Health, the Second Affiliated Hospital Zhejiang University School of Medicine Hangzhou China; 2 Department of Epidemiology Harvard T.H. Chan School of Public Health Boston, MA United States; 3 Division of General Internal Medicine and Primary Care Department of Medicine Brigham and Women’s Hospital Boston, MA United States; 4 Thrust of Data Science and Analytics Hong Kong University of Science and Technology Guangzhou China; 5 Division of Pharmacoepidemiology and Pharmacoeconomics, Department of Medicine, Brigham and Women’s Hospital Harvard Medical School Boston, MA United States

**Keywords:** COVID-19, natural language processing, drugs, social media, pharmacovigilance, public health

## Abstract

**Background:**

While the COVID-19 pandemic has induced massive discussion of available medications on social media, traditional studies focused only on limited aspects, such as public opinions, and endured reporting biases, inefficiency, and long collection times.

**Objective:**

Harnessing drug-related data posted on social media in real-time can offer insights into how the pandemic impacts drug use and monitor misinformation. This study aimed to develop a natural language processing (NLP) pipeline tailored for the analysis of social media discourse on COVID-19–related drugs.

**Methods:**

This study constructed a full pipeline for COVID-19–related drug tweet analysis, using pretrained language model–based NLP techniques as the backbone. This pipeline is architecturally composed of 4 core modules: named entity recognition and normalization to identify medical entities from relevant tweets and standardize them to uniform medication names for time trend analysis, target sentiment analysis to reveal sentiment polarities associated with the entities, topic modeling to understand underlying themes discussed by the population, and drug network analysis to dig potential adverse drug reactions (ADR) and drug-drug interactions (DDI). The pipeline was deployed to analyze tweets related to the COVID-19 pandemic and drug therapies between February 1, 2020, and April 30, 2022.

**Results:**

From a dataset comprising 169,659,956 COVID-19–related tweets from 103,682,686 users, our named entity recognition model identified 2,124,757 relevant tweets sourced from 1,800,372 unique users, and the top 5 most-discussed drugs: ivermectin, hydroxychloroquine, remdesivir, zinc, and vitamin D. Time trend analysis revealed that the public focused mostly on repurposed drugs (ie, hydroxychloroquine and ivermectin), and least on remdesivir, the only officially approved drug among the 5. Sentiment analysis of the top 5 most-discussed drugs revealed that public perception was predominantly shaped by celebrity endorsements, media hot spots, and governmental directives rather than empirical evidence of drug efficacy. Topic analysis obtained 15 general topics of overall drug-related tweets, with “clinical treatment effects of drugs” and “physical symptoms” emerging as the most frequently discussed topics. Co-occurrence matrices and complex network analysis further identified emerging patterns of DDI and ADR that could be critical for public health surveillance like better safeguarding public safety in medicines use.

**Conclusions:**

This study shows that an NLP-based pipeline can be a robust tool for large-scale public health monitoring and can offer valuable supplementary data for traditional epidemiological studies concerning DDI and ADR. The framework presented here aspires to serve as a cornerstone for future social media–based public health analytics.

## Introduction

The emergence of the COVID-19 pandemic has induced an immediate need for effective pharmacotherapies. While the development and application of such therapies are critically important, they are also influenced by an array of political, economic, and social factors. Meanwhile, an overabundance of drug-related information during the COVID-19 pandemic has rapidly proliferated across social media platforms, drawing significant attention from governments and health organizations. This phenomenon, referred to as an “infodemic,” has exacerbated the pandemic’s impact, caused additional harm to individuals, and undermined the effectiveness and sustainability of the global health system [1,2]. For example, public pronouncements by high-profile figures, such as former US President Donald Trump’s endorsement of hydroxychloroquine, have led to its irrational use and consequential public health crises [3]. Traditional pharmacovigilance mechanisms, reliant on clinical trials and formal reporting systems like MedWatch and DrugBank [4-6], offer valuable but lagged information. These traditional approaches are plagued by inefficiencies, reporting biases, and a lack of timeliness, thereby lacking comprehensive coverage of the population’s sentiments and experiences [7-10].

In this context, real-time public comments on pharmacotherapies such as medications on social media provide a valuable resource for complementing research on drug use or repositioning for the COVID-19 pandemic. In addition to the fast accessibility, timeliness, and comprehensive population coverage, social media can also supply real-world evidence on how people respond to different drugs, thus helping researchers mine novel drug potency or side effects [11-13]. Social media also offer data on drugs not typically included in pharmacovigilance datasets, such as over-the-counter drugs [14], herbal remedies [15], and other nontraditional treatments [16]. However, the sheer volume and noise in social media data require robust computational methodologies for effective analysis [17].

Natural language processing (NLP) technologies offer a solution to these challenges. Earlier studies, such as the study conducted by Aramaki et al [18] in 2011, demonstrated that Twitter (subsequently rebranded X) data could be mined to monitor influenza outbreaks using machine learning and rudimentary NLP techniques. Contemporary research in this domain has benefitted immensely from technological advancements, such as deep-learning–based NLP tools specifically for analyzing social media data [19-22]. These have made it increasingly feasible to understand large volumes of colloquial, noisy text for the extraction of meaningful insights on public health.

Substantial efforts such as topic modeling and sentiment analysis have been made to analyze pharmacotherapy-related topics during the COVID-19 pandemic. Notably, existing research lacked of data-driven pipeline with state-of-the-art NLP tools and other big data analysis techniques [23-27] or just involved longitudinal data with a small time span [28]. There is still a gap in how to automatically and accurately extract drug information through social media data for longitudinal monitoring of the drug infodemic.

To address these gaps, this study uses NLP methodologies and network analysis for an extensive assessment of COVID-19 drug-related discourse on social media. We contribute to the existing literature in several ways:

(1) Using deep learning methodologies for named entity recognition (NER), thereby reducing the false positives associated with traditional keyword matching.

(2) Re-examining public sentiments and concerns regarding COVID-19 medications, using target sentiment analysis (TSA) and topic modeling.

(3) Conducting a comprehensive assessment of adverse drug reactions (ADR) and drug-drug interactions (DDI) through network analysis techniques.

We demonstrate that our integrated NLP pipeline can serve as a robust framework for extracting and analyzing drug-related information, thereby enhancing the scope and effectiveness of social media–based pharmacotherapy analysis.

## Methods

### Overview

**Figure 1:**
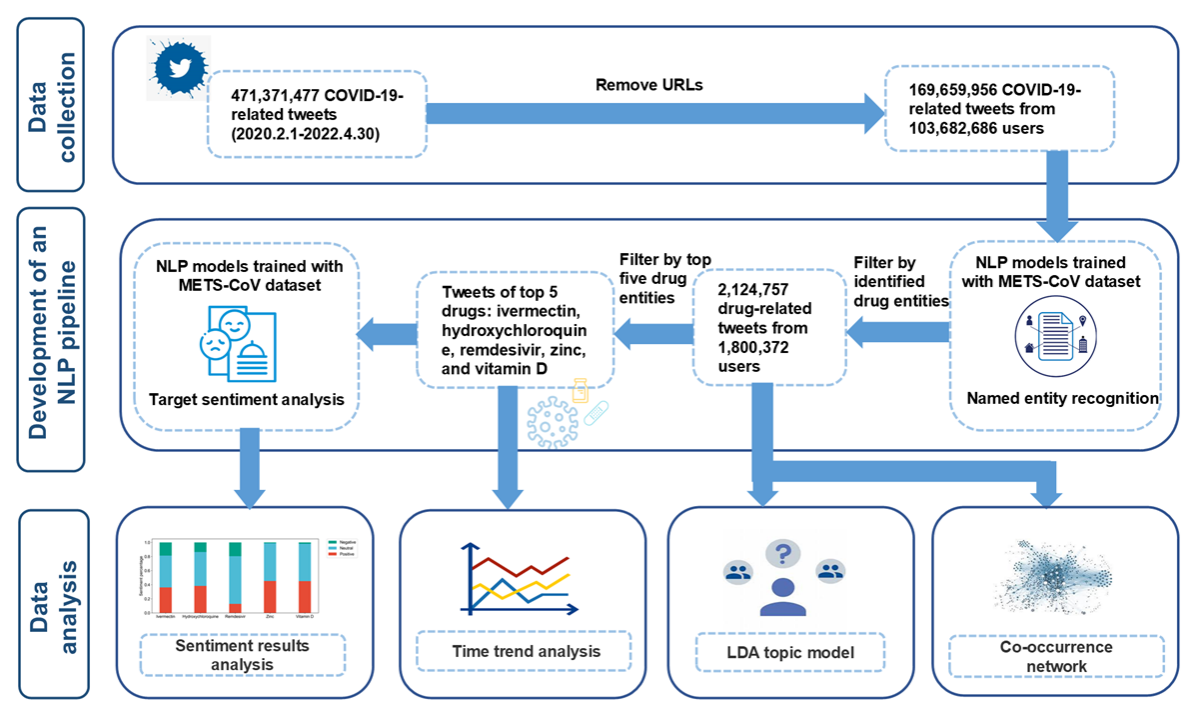
Workflow of drug analysis with natural language processing on Twitter. LDA: latent Dirichlet allocation; METS-CoV: Medical Entity and Targeted Sentiment on COVID-19 Related Tweets; NLP: natural language processing; NER dataset containing medical entities and targeted sentiments from COVID-19–related tweets.

As shown in Figure 1, the study workflow is organized into three primary stages: data collection, development of an NLP pipeline, and subsequent data analysis using the constructed pipeline. Initially, we curated a dataset of English tweets related to the COVID-19 pandemic. After a preprocessing phase that excluded tweets with URLs, an NLP pipeline was developed to extract and normalize the drugs and symptoms mentioned in these tweets. Finally, we examined the time trends of drug mentions, public sentiment, and discussion topics toward drugs, as well as the co-occurrence network of drug-drug and drug-symptom pairs [22].

### Data Collection and Preprocessing

COVID-19–related tweets from February 1, 2020, to April 30, 2022 were downloaded using Twitter’s application programming interface (API) through unique tweet IDs, which were obtained from a public dataset provided by Chen et al [29]. Due to the privacy restrictions of Twitter data, the raw tweets were not publicly available and could only be shared by tweet ID. Therefore, we downloaded tweets by Twitter API based on the provided tweet IDs. The downloaded data included full tweet texts and corresponding metadata such as timestamps and user information. Tweets containing URLs were excluded from the analysis, as they often only contained summaries or quotations of the original tweet. The data collection process adhered to Twitter’s privacy and data use management policies.

### NLP Pipeline Development

The NLP pipeline consists of 4 principal modules: NER, TSA, topic modeling, and drug network analysis. For the NER and TSA modules, we leveraged state-of-the-art models developed in our previous work “Medical Entity and Targeted Sentiment on COVID-19 Related Tweets (METS-CoV)” [22]. Details on model construction can be found in Figure S1 in the Multimedia Appendix 1.

### Named Entity Recognition and Normalization

The NER model aims to extract drug entities from tweets. The model we developed, CT-BERT-NER (COVID Twitter with Bidirectional Encoder Representations from Transformers for Named Entity Recognition) [22], was constructed using the COVID-Twitter-BERT (CT-BERT), a widely adopted language model pretrained on 160 million COVID-19–related tweets. CT-BERT-NER was trained on the entire training set of the NER subset of METS-CoV [22]. Upon evaluation, it showed *F*_1_-scores of 86.35% for drug entity recognition and 81.85% for symptom entity recognition on the corresponding test set, respectively [22]. We used the model trained on all entity types (ie, disease, drug, symptom, vaccine, person, location, and organization) instead of on drug entities only to enable the nuanced differentiation of drug entities from other types of entities.

To standardize colloquial expressions of drugs among the extracted entities, we manually searched Wikipedia for NER-identified drug entities with a frequency of more than 1000 to map colloquial drug expressions and their standardized concepts (ie, drug trade names, chemical names, and generic names). We conducted an accuracy assessment using a random sample of 100 tweets for each of the top 5 most frequently mentioned drugs and symptoms, as identified through 2 methods: NER combined with lexicon-based extraction (NER+lexicon) and lexicon-based extraction alone, with a total of 1000 tweets being manual review. Our results demonstrated that the NER+lexicon method achieved an accuracy rate of 97.8%, significantly surpassing the 89% accuracy achieved by the lexicon-only approach (*χ*^2^_1_=61.4, *P*<.001). Further details on this comparison are available in Table S1 in Multimedia Appendix 1.

### Targeted Sentiment Analysis

The TSA module is designed to analyze users’ sentiments toward specific drug entities within tweets. Inspired by BERT-SPC [30], we first concatenate the original tweet and the identified drug entity, separated by 1 special token “[SEP],” to form a combined tweet-entity sentence. The tweet-entity sentence is then fed into a pretrained language model to capture semantic features, which are subsequently passed to a linear layer for 3-class sentiment prediction (positive, neutral, or negative). Notably, instead of using the original BERT model, we used CT-BERT, which has been further trained on 97 million COVID-19–related tweets. This adaptation enhances its understanding of COVID-19–related tweet data [22]. Therefore, although there existed several sentiment-specific embeddings and pretrained models [31-34], we chose the pretrained CT-BERT model as it was trained to understand COVID-19–related tweets. On the TSA test set of METS-COV, the model achieved an *F*_1_-score of 62.67% and an accuracy rate of 75.07% across 4 entity types: person, drug, disease, and vaccine. For our own TSA study, we randomly selected 100 drug-related tweets and assessed their emotional orientation toward drug entities using both model predictions and manual review by a researcher with medical expertise. The results indicated that the model’s accuracy, when compared to manual review, was 77% (77/100), aligning closely with the TSA model’s original accuracy of 75.07%. Furthermore, both our hand-labeled fine-tuned dataset (METS-CoV) and the final applied dataset (169,659,956 drug-related tweets) were derived from the same source, ensuring the reliability and credibility of the predictions.

### Topic Model Analysis

To discern prevailing public interests in the most discussed drugs, we implemented latent Dirichlet allocation (LDA) for topic modeling, using the LdaModel function from the Gensim package [35]. Topic numbers were determined based on conventional evaluation metrics, including low perplexity [36] and high coherence scores [37]. Detailed methodologies are delineated in Figure S2 in Multimedia Appendix 1.

### Drug Network Analysis

To illustrate potential relationships among drugs, we constructed a drug network analysis module to generate incidence matrices according to a previous study [38] and visualize co-occurrence networks using Gephi [39] and ForceAtlas2 algorithm [40]. For enhanced comprehensiveness, we incorporated a variant supported by the Anatomical Therapeutic Chemical classification system (ATC) [41], in addition to the Gephi-based visualization. In addition, we used the NER model to extract symptom entities and normalize them through a presummarized lexicon list [42] to extend our analysis to drug-symptom networks. The constructed networks feature nodes represented drugs (Table S3 in Multimedia Appendix 1) or symptoms (Table S4 in Multimedia Appendix 1). Node sizes displayed node degrees (ie, the number of linked entities). Edge weights denoted the cosine similarity score of 2 linked nodes. As our focus is not on causal relationships but rather on the interplay between entities, we used undirected graphs and semantic cosine similarity [43] as the distance metric just as we did in the previous work [38]. Cosine similarity is a widely implemented metric in information retrieval and related studies [44]. In our study, each drug or symptom entity can be represented as a vector, with each dimension of the vector corresponding to 1 tweet text. Details for calculation can be found in Methods in Multimedia Appendix 1.

### Pipeline Deployment

Upon completion of the NLP pipeline, we proceeded to its deployment on the preprocessed dataset of COVID-19–related tweets. We first applied the NER and normalization module on the preprocessed dataset (ie, removing URLs) to extract and standardize drug entities to drug concepts. Then we filtered the preprocessed COVID-19–related tweets dataset to get the drug-related tweets dataset according to these drug concepts. Following this standardization, we conducted a distributional analysis of drug mentions to discern time trends, thereby capturing the evolving popularity of these drugs. We also gather related news and the trend of weekly new COVID-19 cases to show a more holistic view of the shift in drug popularity over time. For clarity and simplicity, we only illustrate the top 5 most discussed drugs.

Subsequently, we used the TSA model for drug-related tweets of the top 5 drugs mentioned above to assign each drug entity a sentiment type. To gain a deeper understanding, we also conducted a time-trend analysis on the positive and negative tweets for the 5 drugs and visualized the results. Building upon our understanding of public sentiment, we turned to topic modeling via LDA in all drug-related tweets to explore the thematic concentrations in the discourse surrounding drugs. The model yielded the 20 most probable keywords and bigrams for each identified topic, enabling us to summarize the primary themes. We further analyzed the topic distribution associated with each of the top 5 drugs.

Finally, we constructed co-occurrence networks for drug-drug and drug-symptom interactions to provide a relational overview that complements our earlier analyses. All 67 drugs with more than 1000 mentions and 69 symptoms with more than 250 mentions over time were included in the analysis. Meanwhile, we also zoomed in to analyze the 5 most-discussed drugs.

### Statistical Analysis

The chi-square test was used to compare the accuracy differences between NER combined with lexicon and lexicon-based only. We used Python software (version 3.8) to conduct the statistical analyses and chose a *P* value of .001 as the statistically significant threshold.

### Ethical Considerations

Ethical approval for this study was granted by the Institutional Review Board of School of Public Health, Zhejiang University (ZGL202201-2).

## Results

### Data Summary and Trends of Drug Mention Tweets

This study used a dataset consisting of 471,371,477 COVID-19–related tweets in English, which were collected between February 1, 2020, and April 30, 2022. After excluding tweets containing URLs, the final dataset used for this study consisted of 169,659,956 (36.0%) tweets from 103,682,686 users. Using CT-BERT-NER, we identified 2,124,757 drug-related tweets from 1,800,372 unique Twitter users, accounting for approximately 1.25% of the raw COVID-19–related tweets dataset. Table S2 in Multimedia Appendix 1 provides more detailed statistical results of the medical entity recognition.

**Figure 2:**
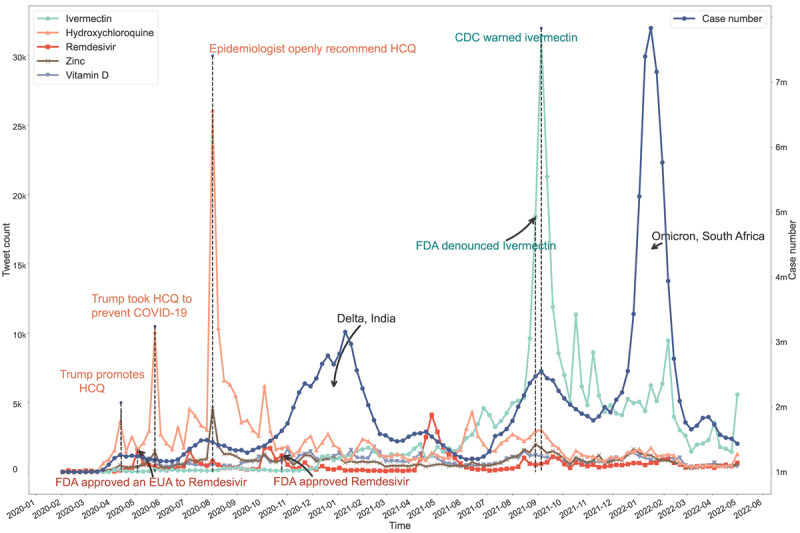
Weekly popularity trends of the top 5 most-mentioned drugs on Twitter examined with COVID-19–related tweets collected between February 1, 2020 and April 30, 2022. The left Y-axis represents the total number of tweets for each drug in a given week (unit: thousand tweets). The right Y-axis represents the weekly new case count (unit: million cases). CDC: Centers for Disease Control and Prevention; FDA: Food and Drug Administration; HCQ: hydroxychloroquine.

Table S3 in Multimedia Appendix 1 presents the 67 most frequently mentioned drugs, each with an occurrence exceeding 1000 times. The most frequent taxonomies are ATC [41] N (nervous system drugs) and J (anti-infective drug). We ranked the total occurrence of all drugs and identified the top 5 most-mentioned drugs: ivermectin, hydroxychloroquine, remdesivir, zinc, and vitamin D to visualize their weekly time trends. Figure 2 presents these temporal trends. The new case counts were collected from the World Health Organization (WHO) [37] on a weekly basis, beginning on February 1, 2020. Given that the dataset is confined to English-language tweets, the scope of new case counts was likewise restricted to the top 4 English-speaking nations with the highest Twitter activity: the United States, the United Kingdom, the Philippines, and Canada [38].

Among the 5 drugs, the public focused mostly on repurposed drugs (ie, hydroxychloroquine and ivermectin), followed by daily supplements (ie, zinc and vitamin D). The only officially approved drug among the 5, remdesivir, received the least attention. The frequency of discussion of hydroxychloroquine and ivermectin fluctuated significantly across time, which seemed to be related to relevant news events or policies (marked in Figure 2). In the early stage of the pandemic, drug-related discussions focused on hydroxychloroquine, with 2 prominent peaks occurring on May 24, 2020, and August 2, 2020. Discussion of ivermectin began to increase in the later stages of the pandemic, with only 1 prominent peak located on September 5, 2021. In contrast, remdesivir received the least public attention, which increased only sporadically throughout the pandemic, with no apparent pattern and a much lower peak on May 3, 2020. As supplements to COVID-19 treatments, vitamin D and zinc elicited much less public interest than ivermectin and hydroxychloroquine, with no significant outbreaks or visible patterns.

### Changes in Sentiment for Five Most Frequent Mentioned Drugs

**Figure 3:**
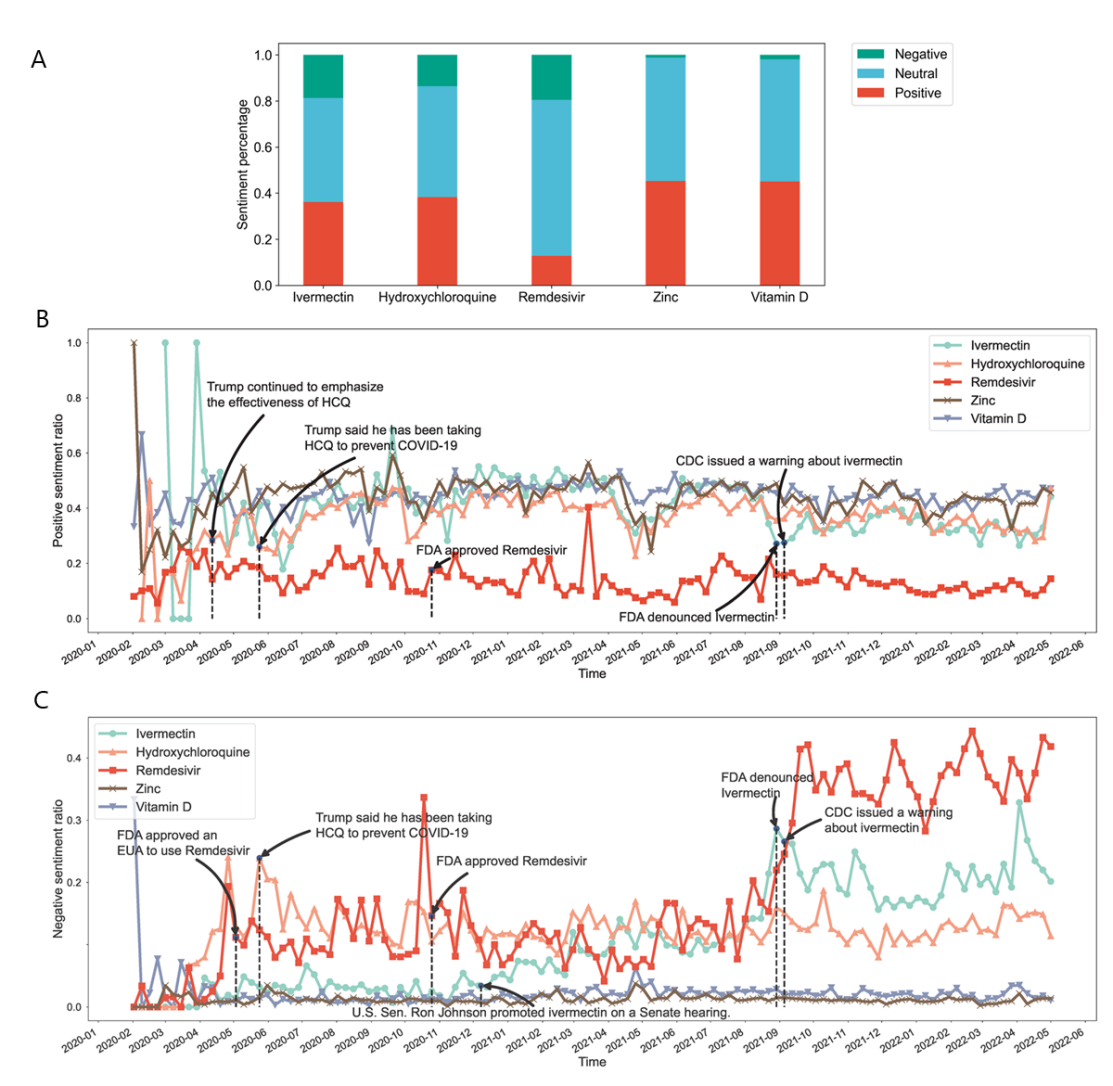
Sentiment analyses of the 5 top-discussed drugs from February 1, 2020, to April 30, 2022, grouped according to their polarity, including (A) sentiment distribution, (B) weekly ratio of positive tweets, and (C) weekly ratio of negative tweets. The denominator of the percentage was the entities with sentiment. CDC: Centers for Disease Control and Prevention; EUA: emergency use authorization; FDA: Food and Drug Administration; HCQ: hydroxychloroquine.

We calculated the sentiment proportion for the 5 drugs and the weekly time trends of positive and negative tweets. Figure 3A shows the visualization of the overall attitude proportions. The public tended to hold positive and neutral attitudes toward the repurposed drugs, ivermectin and hydroxychloroquine. The immune supplements, zinc and vitamin D, were frequently mentioned with positive sentiments. The only COVID-19 drug approved by the Food and Drug Administration (FDA), remdesivir, received the lowest positive attitude, far lower than those of the other drugs.

Figures 3B and 3C present weekly trends of tweets expressing positive and negative attitudes, respectively. The major turning points of the trends tend to coincide with new government policies, major social events, and research findings. The criticism of remdesivir (Figure 3C) and ivermectin increased over time since September 2021, and the turning point for remdesivir came at almost the same time as emerging studies showing that the drug is ineffective [45] and has severe side effects [46-48]. For ivermectin, public sentiment was associated with announcements of health authorities and celebrity effects. For example, the FDA denouncing the use of ivermectin for COVID-19 on August 29^t^, 2021 had simultaneously increasing negative discussions.

### Topic Distributions of Drug-Mentioned Tweets

**Figure 4:**
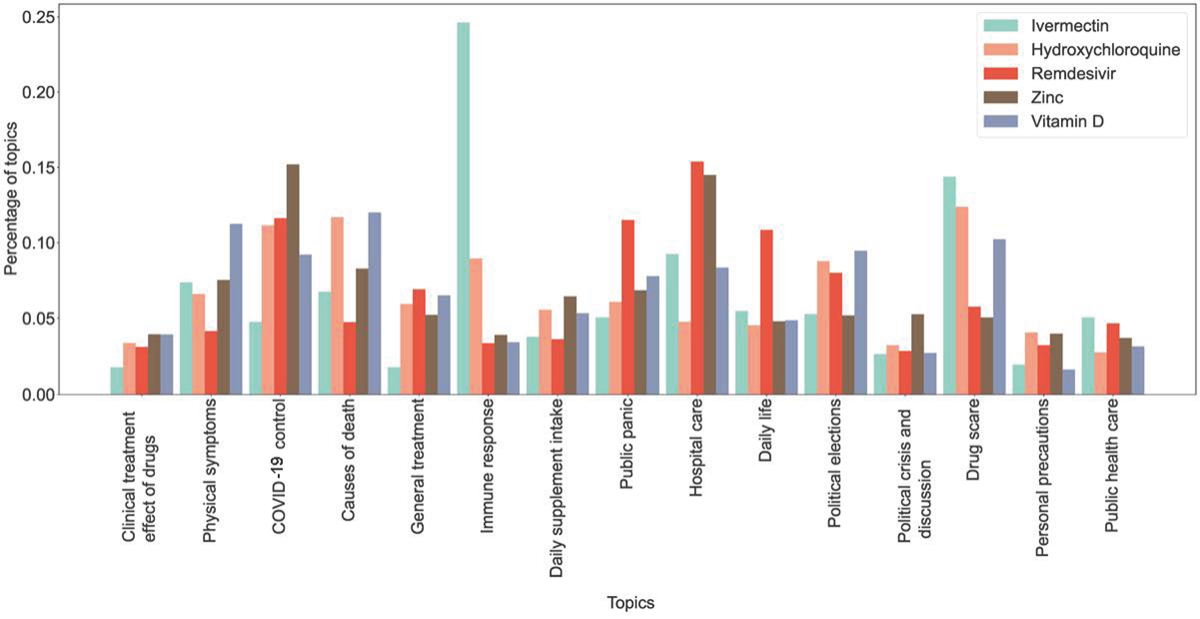
Topic distribution of 5 top-discussed drugs.

We applied the LDA topic model to all 2,124,757 drug-related tweets and obtained 15 general topics based on their relatively high topic coherence scores and low confusion levels (further discussed in Figure S3 in Multimedia Appendix 1). We displayed the corresponding top 20 most likely keywords in Table 1 and assigned a theme for each topic from these keywords. The topic “clinical treatment effect of drugs” included 288,967 related tweets and dominated the discussions, accounting for 13.6% of all related tweets. In addition, 251,571 (11.84%) were related to “physical symptoms,” whereas 220,125 (10.36%), 197,177 (9.28%), 174,868 (8.23%), 172,955 (8.14%), and 154,470 (7.27%) were related to “COVID-19 control,” “causes of death,” “general treatment,” “immune response,” and “daily supplement intake.” In addition to the overall topic summary, we explored the distribution of the 15 topics for the 5 drugs. Figure 4 shows a visualization of the distribution. For ivermectin, the prominent theme was “immune response.” In contrast, discussions of remdesivir centered on “hospital care.” Hydroxychloroquine received relatively even attention among the 3 topics “causes of death,” “drug scare,” and “COVID-19 control.” Vitamin D was frequently mentioned in tweets about “daily life,” and the main topics about zinc focused on “hospital care” and “COVID-19 control.”

**Table 1:** Topic model on drug-related tweets.

Topic	Keywords	Example	Number and Percentage of related tweets, n (%)
Clinical treatment effect of drugs	treatment, covid, study, drug, effective, trial, hydroxychloroquine, prove, safe, covid, evidence, prevent, clinical, vaccine, recommend, cheap, infection, continue, antiviral, efficacy	“@USER^a^ Ivermectin is pretty safe but the evidence for it being efficacious against SARS-CoV-2 is lacking.”	288,967 (13.60%)
Physical symptoms	day, test, symptom, week, covid, positive, feel, month, steroid, bad, start, med, ago, sick, time, recover, antibiotic, hour, fine, insulin	“When I had covid, it was mild fever for a day, &amp; it was gone”	251,571 (11.84%)
COVID-19 control	cure, vaccine, spread, control, lie, people, drug, covid, push, approve, hydroxychloroquine, claim, force, medium, ban, talk, science, government, experimental, president	“@USER^a^ I agree with you re lockdown. Just not on HCQ and vaccines.”	220,125 (10.36%)
Causes of death	death, people, die, covid, kill, reason, heart, drug, cancer, cocaine, dead, trust, epidemic, rate, bad, create, attack, sound, result, fentanyl	“@USER^a^ Of course, if they died 30 minutes after taking fentanyl but had a positive covid test, guess what their official cause of death is listed as?”	197,177 (9.28%)
General treatment	doctor, treat, patient, steroid, risk, covid, covid, severe, infection, blood, pill, medication, antibiotic, receive, prescribe, illness, lung, hospitalize, prescription, aspirin	“@USER^a^ migraines have a very specific causality (i had them for like 20 years), I wonder if the Covid version is one? -- I would try warm compress, NSAIDs, and maybe nasal irrigation with like a neti pot”	174,868 (8.23%)
Immune response	virus, system, body, immune, corona, zinc, fight, antibody, cure, immunity, bleach, deficiency, cell, response, kill, covid, inject, injectingdisinfectant, human, boost	“In addition, mAbs have been shown to improve survival in patients hospitalized with COVID-19 who have not mounted their own immune response.”	172,955 (8.14%)
Daily supplement intake	vitamin, people, level, covid, eat, healthy, cold, flu, catch, protect, food, hand, low, stay, vit, survive, bad, chance, common, worry	“@USER^a^ regularly take vitamins and a Vitamin D supplement. I started taking the Vitamin D supplement because I wasn't going outside as much at the start of the pandemic. As soon as started taking the Vitamin D supplement, my blood work started to improve.”	154,470 (7.27%)
Public panic	live, save, pandemic, life, start, lockdown, real, people, buy, time, basicallystart, money, watch, steroid, hit, hard, normal, deadly, cough, break	“@USER^a^ waste of time, politicians have organised orgy’s with cocaine, male &amp; female hookers, during lockdown! like they give a fuck about a petition hahaha”	149,583 (7.04%)
Hospital care	patient, care, hospital, remdesivir, treatment, cocktail, covid, covid, injection, require, oxygen, admit, medical, lead, ventilator, pay, provide, health, remove, source	“Both hospitalized and treated immediately with Oxygen &amp; Remdesivir for covid @ the same time. Both went into heavy psychosis.”	118,136 (5.56%)
Daily life	drink, stay, hear, lose, family, wait, water, love, leave, pandemic, friend, daily, close, rest, hope, lot, drop, head, play, time	“Interesting situation. Got a call from a Mom, Family of 4 lives in a house. Son and her drinks SOULTOX everyday. Daughter and Dad don't. Dad got Covid, then daughter. Mom and Son tested negative 4xs over the 2 wks. Everyone is vaxed. Now she makes everyone drink SOULTOX now.”	97,952 (4.61%)
Political elections	dem, trump, fake, nursinghome, news, pandemic, free, vote, ill, school, election, economy, release, access, truth, guy, forget, sense, deny, hoax	“@realDonaldTrump Hey Captain Covid I VOTED FOR JOE BIDEN AND KAMALA HARRIS! The steroids and tranquilizers are making you more batshit crazy than usual. You should go back to the hospital.”	71,179 (3.35%)
Political crisis and discussion	trump, people, person, infect, dangerous, true, covid, condition, shit, happen, stock, send, woman, idea, stupid, destroy, contract, completely, potus, tablet	“@realDonaldTrump @USER^a^ At least Putin just poisons his political enemies -- Trump wants all Americans to drink poisons such as hydroxychloroquine and oleandrin -- there just aren't enough dead Americans from COVID-19 for Trump -- he wants to kill more with poison -- Trump is a quack ...”	70,117 (3.30%)
Drug scare	country, low, rate, dexamethasone, cost, covid, drug, recovery, black, base, reduce, sell, supply, explain, mortality, expect, increase, panic, produce, improve	“Once the pandemic hit, stores really upped the prices of aloe vera leaves like i wouldn’t notice smh”	60,981 (2.87%)
Personal precautions	mask, wear, medicine, social distance, add, avoid, business, plasma, measure, spray, confirm, skin, drive, advise, campaign, practice, oil, wash hand, air, hourly	“@USER @USER @USER^a^ Try working on your immune system. Covid depletes zinc and vitamin D. Try working from prevention instead of fear.”	48,444 (2.28%)
Public health care	report, support, health, public, supplement, question, kid, child, datum, stage, issue, american, answer, term, prophylactic, mental, phase, safety, concern, inflammation	“@USER^a^ Then, even hcq, zinc, and zithromax had a estimated 50% success and the CDC and WHO said it didnt, so it shouldnt be used. Imagine half of the death tolls because of at least TRYING something instead of shitcanning it just because you are an “authority” that hates Trump.”	48,232 (2.27%)

^a^Username and other sensitive information were masked off using @USER. Public figures such as @realdonaldtrump are shown in their usernames.

### Co-Occurrence Networks

**Figure 5:**
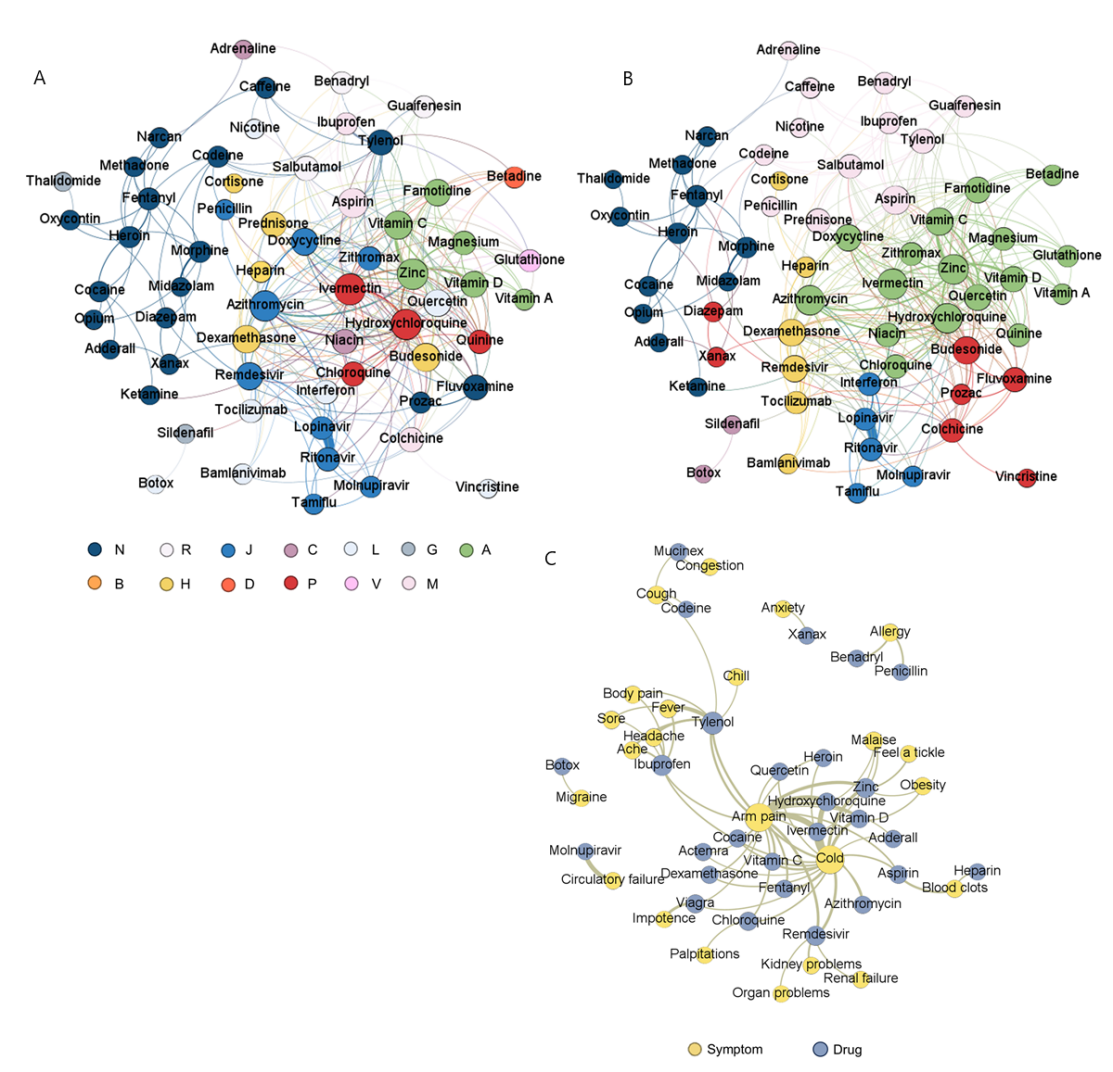
Visualization of drug-related co-occurrence networks by Gephi, including (A) drug-drug associations based on Gephi clustering (τ=0.005), (B) drug-drug associations based on ATC (τ=0.005), and (C) drug-symptom associations (τ=0.05). The color dots on the lower right of the figure represent the ATC categories for (B). ATC: Anatomical Therapeutic Chemical classification system.

We visualized the co-occurrence network for drug-drug and drug-symptom relations in Figure 5. The nodes represented either drugs (as shown in Table S3 in Multimedia Appendix 1) or symptoms (as shown in Table S4 in Multimedia Appendix 1). The size of each node corresponded to its degree, which referred to the number of connections it has. The weights of the edges indicated the cosine similarity score between two connected nodes.

### Drug-Drug Network

The origin drug-drug network contained 67 drugs (nodes) with more than 1000 mentions and 1103 relations (edges) among them. A predefined similarity threshold (τ) was established to only visualize relationships with substantial co-occurrence, as measured by cosine similarities exceeding τ. After filtering it with a τ of 0.005, 62 drugs and 317 relations remained in the network. By using the Fast Unfolding (Louvain) algorithm built in Gephi for modularity classification [49], the drugs were clustered into 5 categories and were colored in Figure 5A. The same network with drugs colored by ATC classification (12 types) was shown in Figure 5B for comparison. Drugs in the same group are denoted with the same color. Both figures share similar clustering characteristics, especially in psychotropic drugs ATC-N (Anatomical Therapeutic Chemical classification system, psychotropic drugs; eg, fentanyl, opium, and morphine) and anti-infectious agent ATC-J (Anatomical Therapeutic Chemical classification system, anti-infectious agents; eg, lopinavir, ritonavir, and azithromycin). However, drugs in the ATC-P (Anatomical Therapeutic Chemical classification system, antiparasitic drugs) group (ie, ivermectin, hydroxychloroquine, quinine, and chloroquine) are clustered with the ATC A group in Figure 5A. The reason may partially lie in the fact that most parasites are intestinal [50], so most people who need to take antiparasitic drugs (ie, ATC-P drugs) often present concomitant digestive manifestations [51], thus necessitating the use of digestive medications (ie, ATC A drugs), therefore the 2 drug groups are closely related. Association between some of the significant drug-drug pairs like 2 HIV protease inhibitors ritonavir and lopinavir has been widely studied [52]. In addition, through the co-occurrence network, we observed several unusual drug pairings, such as midazolam and morphine, salbutamol and prednisone, and zinc and quinine. These strong co-occurrences suggest potential unexplored synergistic effects, adverse reactions, or other public health concerns that warrant further investigation. For instance, we noted a distinct correlation between morphine and midazolam, drugs not typically combined in direct COVID-19 treatment. An analysis of all 376 tweets mentioning both drugs revealed that most discussions focused on end-of-life management for patients with COVID-19 and on conspiracy theories about the intentional misuse of these drugs, leading to deaths attributed to causes other than COVID-19 infection.

### Drug-Symptom Network

The original drug-symptom network had 136 nodes (ie, 69 drugs and 67 symptoms) and 3099 edges. After filtering by τ of 0.05, 50 nodes and 71 edges remained and are shown in Figure 5C. We observed that the edges often represented symptoms and corresponding treatments, such as Tylenol for fever medication, suggesting the reliability of our association network. We also observed some side effect relations, such as remdesivir to acute kidney failure [46] and some novel associations receiving no clinical investigation like molnupiravir to circulatory failure, cocaine to chest cold, and vitamin D to malaise. We visualized the top 10 closest drugs and symptoms with co-occurrence relationships to the 5 drugs under investigation (Figure S4 in Multimedia Appendix 1). These networks revealed the great relevance between hydroxychloroquine, ivermectin, and azithromycin from each other. Furthermore, remdesivir was also significantly associated with dexamethasone and tocilizumab.

## Discussion

### Principal Results

Leveraging new advances in NLP, we constructed a pretrained language model driven by the drug entity recognition model and a new targeted sentiment analysis model for the polarity prediction of target drugs. Based on over 2 years of relevant data, our comprehensive NLP pipeline demonstrates advanced accuracy and completeness in collecting and analyzing data for social media-based drug studies. Our NER model identified the top 5 most-discussed drugs and sentiment and topic analysis revealed that public perception concerning these drugs was predominantly shaped by celebrity endorsements, media hot spots, and governmental directives rather than empirical evidence of drug efficacy. Furthermore, network analysis identified emerging patterns of DDI and ADR (ie, molnupiravir to circulatory failure) that could be critical for public health surveillance like better safeguarding public safety in medicines use. Our pipeline is open-sourced and it can serve as a comprehensive tool to enhance drug safety control, provide crucial guidance for formulating drug usage policies, and support public health decision-making after the outbreak of infectious diseases.

Compared with traditional pharmacovigilance research, the study of drug-related information on social media exhibits distinctive characteristics and advantages. Social media platforms offer real-time and immediate data, enabling the rapid reflection of drug usage patterns and patient feedback, facilitating the prompt identification of potential risks and benefits [53-55]. Furthermore, social media captures the viewpoints and experiences of patients, thus furnishing critical insights for the formulation of patient-centered care [56,57]. For example, understanding patient’s preference for drugs and disease burden can improve drug development strategies, enabling pharmaceutical companies to better focus on specific drugs that meet patient needs and preferences [58]. In contrast to previous COVID-19 social media studies [59-62], this work extracted more rigorous data covering a more extended study period and identified the five most discussed drugs to be investigated through a fully data-driven method. The substantial volume of social media data allows for large-scale real-time dynamic analysis, and it also covers a broader population than electronic health records, which are confined to hospitalized individuals and have restricted access [63]. Social media datasets could also provide large-scale samples for the detection of rare events and the examination of specific population responses, which are challenges in electronic health records–based analysis.

Sentiment analysis on drugs can highlight patient misconceptions and disagreements about a specific medication, enabling pharmaceutical companies and public health agencies to address public anxiety and reduce misinformation about drugs. Our results confirmed findings from Hua et al [59] that the public concern and polarity for ivermectin and hydroxychloroquine, which received the most social media attention, are highly correlated with emotional and political factors, such as personal political orientation, presidential elections, and conspiracy theories. For instance, there was a surge of approximately 200% in acquisitions of medication alternatives such as hydroxychloroquine within 2 days after the press briefing conducted by Donald Trump on March 19, 2020 [3]. The topic distribution indicated possible effects or side effects of ivermectin on the immune system and the wide in-hospital treatment use of remdesivir, but the sentiment analysis showed most opposing stances toward remdesivir which climbed significantly as the crisis unfolded. It was due to shortages, emergency needs, inefficiency [64], and potential side effects of remdesivir like bradycardia [65], and increased risk of hepatic, renal, and cardiovascular reactions [66,67]. Some people even hyped up on Twitter that remdesivir was approved solely for the purposes of reaping big profits for Anthony Fauci and the democidal cabal that he fronts, bilking the taxpayers of billions, and all while quietly euthanizing an unwitting public. Furthermore, we also found that daily supplements like zinc and vitamin D did not attract much public attention, but their immune-enhancing properties make them significantly more commended by the public than the other three drugs, especially remdesivir.

Analyzing social media data helps identify patterns of drug abuse, adverse reactions, and epidemics, thereby improving health policy planning and resource allocation to address emerging challenges [68,69]. For example, social media plays a pivotal role in addressing drug-related outbreaks and trends, enabling policy makers to respond swiftly and enhance public safety. Its interactive nature fosters direct engagement with the public, allowing policy makers to better understand community needs and concerns. Since public trust in policy makers is critical, for instance, the successful promotion of drugs and vaccines relies heavily on public confidence [70,71], tracking public sentiment through social media in real time enables policy makers to align policies with public attitudes, so as to increase their acceptance and effectiveness. In addition, this approach helps in combating misinformation about drugs and vaccines [72]. For public health agencies, timely monitoring of drug-related concerns on social media is especially crucial when managing new drug candidates during pandemics. Specifically, our pipeline allows for real-time monitoring of public opinion on social media, which can be an important tool for public health agencies and organizations to implement clear communication plans, physical and mental health interventions, and a coordinated emergency response [73,74]. It could also help conduct rapid and dynamic screening of special populations [60] during public health emergencies, enable targeted communication [75], and combat public health misinformation [72,76].

Our work found that Twitter discussion topics of drugs during the COVID-19 pandemic were consistent with relevant studies focusing on non-drug COVID-19–related topics [25,77,78]. Similar to them, this study uncovered public concerns about “public health measures” and “treatment and recovery.” In addition, by focusing on drugs, we discovered new drug-specific concerns, such as “drug panic” and “immune response.” The focus on “drug panic” may reflect societal uncertainty and anxiety about drug use during the epidemic. Understanding these anxieties can be instrumental in enabling mental health professionals and policy makers to take measures to support mental health and implement interventions to alleviate anxiety. Care about the “immune response” may be indicative of public concerns about the immune system, including vaccines and immunotherapies. This can help health agencies better communicate information about vaccinations and immunization support to increase public awareness of immunization.

Many previous studies aimed to detect potential DDI and ADR from social media [79-81] or online literature [82] but largely depended on external vocabulary for keyword-matching and little visualization was performed. This study used advanced pretrained language models to identify drug mentions and classify the corresponding sentiments from social media text, ensuring the accuracy of information extraction and sentiment prediction. As the pretrained language model is the main NLP structure in our pipeline, it can be easily extended by integrating better large language models (LLMs) [83-85] that have a similar deep learning network structure but with larger parameters, given enough computational resources. The visualization module could illustrate associations between drugs, drug-symptoms pairs, and possible clusters or patterns intuitively and clearly, making it easier for researchers to understand and interpret the findings for DDI and ADR [86]. In addition, our co-occurrence network analysis found many widely studied drug-drug and drug-symptom pairs which could verify the reliability of network analysis. The clustering results are consistent with the classification of the general clinic (ie, ATC) to a certain extent, such as the similar clustering characteristics in psychotropic drugs (ie, ATC N) and anti-infectious agent (ie, ATC J), suggesting its potential to capture similarities and associations between drugs. Notably, we also found many drug pairs with not widely examined associations, such as zinc and quercetin. Their complex (Q/Zn) is considered a potential new drug therapy for improving glycemic control and pulmonary dysfunction in diabetes mellitus [87], which needs to be further investigated. We found new drug-related associations, such as rheumatoid drugs (hydroxychloroquine, dexamethasone, etc.) may affect COVID-19 treatment due to drug repositioning. For these novel drug-drug and drug-symptom pairs, researchers interested in further exploration may undertake additional studies, such as cross-study analyses using multiple data sources or more detailed quantitative studies. We expect studies could examine the novel associations and provide more robust evidence in future work. Furthermore, networks of the top 5 drugs revealed the significant associations between them such as the co-medication of ivermectin, hydroxychloroquine, and azithromycin for COVID-19 infection. Our network analysis also indicated the combination of remdesivir and tocilizumab or dexamethasone, and a randomized controlled trial showed their efficacy for the treatment of severe COVID-19 infection [88-90].

In essence, the use of NLP techniques and network analysis in our pipeline to analyze vast amounts of social media data is an emerging research approach in pharmacovigilance [91,92]. It holds immense potential in various areas such as the monitoring of ADR, the analysis of drug usage trends, the prediction of epidemics, and the evaluation of drug treatment effects. This novel method could serve pharmaceutical firms, regulatory agencies, and the health care fields with more precise and timely information to enhance their efforts in safeguarding public health.

### Limitations

Certain limitations apply to this study. First, social media users can’t represent the general population. For example, Twitter users in the United States are younger, more democratic in their political affiliations, and the most prolific 10% of users create 80% of tweets [93] and older people with lower socioeconomic status may have limited access to social media [94], which may result in bias of our observations. The specific events, geographical contexts, and the dynamic nature of social media usage may also influence the observations. Second, although we tried to automate the information extraction with deep learning, we still relied on an empirical lexicon to cluster different concept representations. This allowed us to effectively reduce false positives but not to avoid false negatives. Third, manual checks for symptom recognition suggested that approximately 2%-3% of the tweets may still be false positive (eg, lexical ambiguity like an American fever dream), which would lead to fake associations, despite the combination of rigorous rules and advanced NLP models based on deep learning. Data accuracy, as well as the reliability of the network analysis, are also limited by the authenticity of social media data and the influence of noisy information and misinformation. However, the primary advantage of social media information is its vast scale and timeliness, which offers opportunities for advancing valuable research directions, such as identifying novel drug interactions. Finally, due to the relatively low accuracy of the TSA module (ie, 75.07%), future work should develop more effective NLP solutions to facilitate opinion mining.

### Comparison With Previous Works

Hua et al [59] used BERT models to examine public perceptions of approved and off-label medications for COVID-19 infection and found these perceptions to be heavily skewed by misinformation and biases. However, the study suffered from methodological limitations, including a narrow and subjectively chosen selection of drugs, manual lexicon-based extraction, and a small time span. Similarly, Wu et al [38] made the very first attempt to construct co-occurrence networks to study symptoms during COVID-19 infection, but their technique was solely based on lexicon matching. Both of them relied solely on lexicons for extraction and, as a result, suffered from insufficient accuracy and a lack of generalizability. In contrast, this study combined advanced deep learning models with lexicon-match to improve the accuracy of entity recognition and sentiment analysis, creating a comprehensive and generalized pipeline to streamline information tracking in public health emergencies.

### Conclusion

Our study proposed a pipeline of using social media data and NLP techniques to mine potential drug information, timely track drug-related hot events, facilitate public health stakeholders to conduct reasonable policy enactment, monitor drug public opinion, and avoid malignant events during a public health emergency period. In addition, it can supplement the existing ADR and DDI databases by constructing multiple medical entity co-occurrence networks to provide real-world clues for future research. Our framework applies not only to the COVID-19 pandemic but also to other periods of epidemics or major social events. It can also target other public health care foci such as vaccination.

## Appendix

Multimedia Appendix 1 Additional materials.

## Abbreviations

ADR: adverse drug reactions
API: application programming interface
ATC: Anatomical Therapeutic Chemical classification system
ATC-J: Anatomical Therapeutic Chemical classification system, anti-infectious agents
ATC-N: Anatomical Therapeutic Chemical classification system, psychotropic drugs
ATC-P: Anatomical Therapeutic Chemical classification system, antiparasitic drugs
CT-BERT: COVID-Twitter-Bidirectional Encoder Representations from Transformers
CT-BERT-NER: COVID Twitter with Bidirectional Encoder Representations from Transformers for Named Entity Recognition
DDI: drug-drug interactions
FDA: Food and Drug Administration
LDA: latent Dirichlet allocation
METS-CoV: Medical Entity and Targeted Sentiment on COVID-19 Related Tweets
NER: named entity recognition
NLP: natural language processing
TSA: target sentiment analysis
WHO: World Health Organization

## Data Availability

Due to the privacy restrictions of Twitter, only tweet IDs can be released. Tweet IDs can be obtained from https://github.com/echen102/COVID-19-TweetIDs. The source code and pipeline tutorial of this paper are available at https://github.com/zju-liwanxin/covid-twitter-drug. Datasets and models for NER and TSA models are publicly available at https://github.com/YLab-Open/METS-CoV. All codes are based on Python software (version 3.8) and the NER, and TSA models are developed by PyTorch (version 1.0).
